# Live imaging of osteoclast inhibition by bisphosphonates in a medaka osteoporosis model

**DOI:** 10.1242/dmm.019091

**Published:** 2016-02-01

**Authors:** Tingsheng Yu, Paul Eckhard Witten, Ann Huysseune, Anita Buettner, Thuy Thanh To, Christoph Winkler

**Affiliations:** 1 Department of Biological Sciences, National University of Singapore, Singapore117543; 2 NUS Centre for Bioimaging Sciences (CBIS), Singapore 117557, Singapore; 3 Department of Biology, Ghent University, 9000 Ghent, Belgium

**Keywords:** Osteoporosis, Bone modeling, Remodeling, Homeostasis, Osteoblast-osteoclast coupling, Medaka

## Abstract

Osteoclasts are bone-resorbing cells derived from the monocyte/macrophage lineage. Excess osteoclast activity leads to reduced bone mineral density, a hallmark of diseases such as osteoporosis. Processes that regulate osteoclast activity are therefore targeted in current osteoporosis therapies. To identify and characterize drugs for treatment of bone diseases, suitable *in vivo* models are needed to complement cell-culture assays. We have previously reported transgenic medaka lines expressing the osteoclast-inducing factor receptor activator of nuclear factor κB ligand (Rankl) under control of a heat shock-inducible promoter. Forced Rankl expression resulted in ectopic osteoclast formation, as visualized by live imaging in fluorescent reporter lines. This led to increased bone resorption and a dramatic reduction of mineralized matrix similar to the situation in humans with osteoporosis. In an attempt to establish the medaka as an *in vivo* model for osteoporosis drug screening, we treated Rankl-expressing larvae with etidronate and alendronate, two bisphosphonates commonly used in human osteoporosis therapy. Using live imaging, we observed an efficient, dose-dependent inhibition of osteoclast activity, which resulted in the maintenance of bone integrity despite an excess of osteoclast formation. Strikingly, we also found that bone recovery was efficiently promoted after inhibition of osteoclast activity and that osteoblast distribution was altered, suggesting effects on osteoblast-osteoclast coupling. Our data show that transgenic medaka lines are suitable *in vivo* models for the characterization of antiresorptive or bone-anabolic compounds by live imaging and for screening of novel osteoporosis drugs.

## Abstract (precis)

**Summary:** Live imaging in the medaka, a popular fish model for human bone research, shows that osteoclast inhibition by bisphosphonates triggers an efficient repair of bone defects by redistributed osteoblasts.

## INTRODUCTION

Bone is a highly dynamic tissue that undergoes continuous remodeling to retain its stability and rigidity. The resorption of mineralized bone matrix by osteoclasts concurs with deposition of new bone by osteoblasts. To obtain homeostasis and prevent disequilibrium, the number and activities of various bone cell types need to be tightly coordinated. The concept of a ‘basic multicellular unit’ that underlies bone remodeling implies a feedback between different bone cells (Harada and Rodan, 2003). Accordingly, increased bone resorption also stimulates an increase of bone formation and vice versa. Interactions between osteoclasts and osteoblasts occur during all phases of bone formation and remodeling and are crucial for bone homeostasis (reviewed by Charles and Aliprantis, 2014). In disease conditions, cell communication between osteoblasts and osteoclasts is perturbed. In osteoporosis, for example, this leads to increased osteoclast activity and bone resorption, causing reduced bone mineral density and increased fracture risk. Also, age-related bone loss is associated with significant changes in bone remodeling characterized by decreased bone formation relative to bone resorption, resulting in increased bone fragility (e.g. see Khosla and Riggs, 2005; Manolagas, 2010). In the USA, 55% of people 50 years of age and older have an increased risk to develop osteoporosis, and of the approximately 10 million Americans with osteoporosis, 80% are women (Nanes and Kallen, 2009).

Current osteoporosis therapies aim to increase bone mass by treatment with either bone-anabolic or antiresorptive drugs. Although anabolic therapies are presently limited to intermittent parathyroid hormone (iPTH) treatment, several antiresorptive therapies are available. Among these, bisphosphonates (BPs) are currently the most frequently used drugs in human osteoporosis therapy (Rogers et al., 2011). BPs chelate Ca^2+^ on the bone surface, affect bone metabolism and lead to a general reduction in bone turnover (Jobke et al., 2014). When osteoclasts are in their resorptive phase, a highly acidic microenvironment is created. This facilitates the release of BPs in the resorption lacunae and leads to high local concentrations of BPs that are then internalized into osteoclasts by endocytosis.

Etidronate is a first-generation non-nitrogenous BP that forms cytotoxic ATP analogs after internalization. These block energy metabolism and impair osteoclast function, eventually triggering caspase-mediated apoptosis (Benford et al., 2001; Dominguez et al., 2011; Ebetino et al., 2011). The nitrogen-containing alendronate is a member of the second BP generation that inhibits the mevalonate pathway and sterol biosynthesis and prevents prenylation of small GTPases (Luckman et al., 1998). This disrupts the osteoclast cytoskeleton and prevents formation of a ruffled border, resulting in the loss of resorptive osteoclast function (Ebetino et al., 2011; Jobke et al., 2014; Rogers et al., 2011). Besides blocking osteoclast function and survival, BPs also have a deleterious effect on bone formation in human patients. Long-term use of BPs results in brittle bone and increased risk of bone fractures (Rogers et al., 2011). Therefore, more efficient drugs would aid efforts to alleviate bone deficiencies more specifically, and *in vivo* models are needed to identify and characterize these drugs.

Compared with cell-culture settings, *in vivo* models provide valuable insight into the multicellular networks implicated in bone homeostasis. Zebrafish and medaka have become popular models in bone research (reviewed by Apschner et al., 2011; Mackay et al., 2013). Their almost transparent embryos and larvae allow live imaging at high temporal and spatial resolution during bone modeling and remodeling (Apschner et al., 2014; To et al., 2012). Teleost osteoblasts and osteoclasts share many features with their mammalian counterparts. Like mammals, teleost fish form bone through chondral and intramembranous bone formation and undergo bone remodeling (Witten and Huysseune, 2009). We previously reported generation of transgenic medaka that express fluorescent reporters in bone cells under control of various osteoblast- and osteoclast-specific promoters; this includes osteoblast progenitors (*collagen type 10a1*, *col10a1*; Renn et al., 2013), premature and mature osteoblasts (*osterix*, *osx* and *osteocalcin*, *osc*; Renn and Winkler, 2009) and osteoclasts (*cathepsin K*, *ctsk*; To et al., 2012). We also generated a transgenic line that expresses the osteoclast-inducing factor receptor activator of nuclear factor κB ligand (Rankl) under control of a heat-inducible promoter (To et al., 2012). Induction of Rankl in this system results in ectopic formation of activated osteoclasts. This leads to increased bone resorption and a severe osteoporosis-like phenotype, with drastically reduced mineralization in the vertebral bodies. This unique transgenic model allows *in vivo* visualization of osteoclast formation and osteoblast-osteoclast interaction by live imaging (To et al., 2012).

Bone-anabolic compounds have been tested in fish models in the past (Barrett et al., 2006; Fleming et al., 2005). These early studies established the suitability of fish larvae to assess the effects of compounds on the mineralized skeleton efficiently. In the present study, we used live imaging in medaka to visualize osteoblast and osteoclast behavior in the presence of BPs *in vivo*. We report that BPs efficiently block osteoclast activity and promote bone recovery in this unique transgenic model.

## RESULTS

### Etidronate and alendronate inhibit osteoclast activity in medaka and prevent mineralization loss after Rankl induction

Etidronate and alendronate have previously been shown to maintain skeleton mineralization in a zebrafish model for glucocorticoid-induced osteoporosis, but their effect on bone cells remained unknown (Barrett et al., 2006; Fleming et al., 2005). We used similar BP concentrations to determine the effect of BPs on osteoclast formation and activity in bone transgenic medaka reporter lines. Dose-response studies showed that a concentration of 10 μg/ml for etidronate and 50 μg/ml for alendronate was most efficient to affect osteoclast activity in medaka (see Fig. S1).

**Fig. 1.**
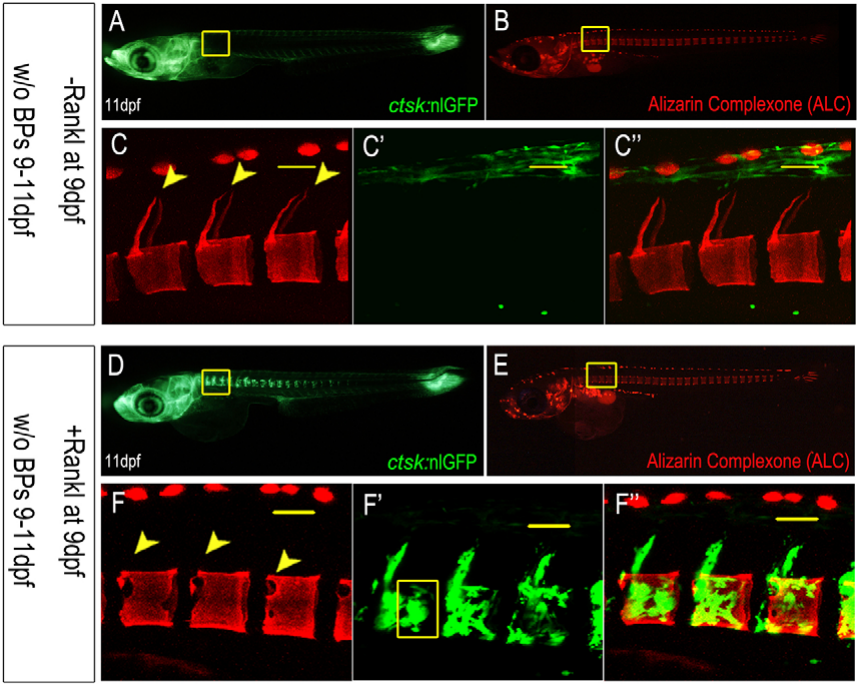
**Osteoclast formation and vertebral body mineralization after Rankl induction.** (A,B) Absence of *ctsk*:nlGFP-expressing osteoclasts in vertebral bodies in *ctsk*:nlGFP larvae at 11 dpf (A) and intact mineralization (B) without Rankl induction. (C-C″) Confocal stack of area boxed in (A,B), showing ALC-stained intact vertebral bodies (C), GFP autofluorescence of pigment cells (C′) and an overlay with GFP (C″). (D,E) Ectopic osteoclast formation at 11 dpf, after Rankl induction at 9 dpf in *rankl*:HSE:CFP/*ctsk*:nlGFP larvae (D) and ALC-stained mineralization (E). (F-F″) Confocal stack of area boxed in (D,E), showing absence of mineralized neural arches and cavities in vertebral centra (arrowheads in F), where active osteoclasts cover the vertebral bodies (overlay in F″). Scale bars: 50 μm.

To test how BP treatment affects osteoclast activity *in vivo*, we analyzed *rankl*:HSE:CFP/*ctsk*:nlGFP double transgenic medaka larvae. Larvae were heat shocked at 9 days postfertilization (dpf) for 1.5 h to induce ectopic osteoclast formation, as described previously (To et al., 2012). The heat shock slightly delayed larval development, as evident by the extent of mineralization in the caudal fin rays. Importantly, however, this delay was identical in non-BP-treated and BP-treated larvae (Fig. S2). Wild-type control larvae or transgenic larvae without Rankl induction develop a mineralized vertebral column at 11 dpf, with mineralized dorsal neural arches and vertebral centra (Fig. 1A-C″). No *ctsk*:nlGFP-expressing osteoclasts are visible in the trunk region in control larvae at this stage (Fig. 1A,C). After heat shock-induced Rankl expression, excessive numbers of ectopic *ctsk*:nlGFP-expressing osteoclasts were found covering vertebral bodies and neural arches (Fig. 1D-F″). Accumulation of these tartrate-resistant acid phosphatase (TRAP)- and CathepsinK (Ctsk)-positive osteoclasts (see To et al., 2012) results in enhanced resorption of mineralized matrix, which consequently leads to the absence of alizarin complexone (ALC)-stained neural arches and large non-mineralized cavities in the notochordal sheath and vertebral centra in 82.2% of the analyzed larvae (*n*=52; arrowheads in Fig. 1F).

**Fig. 2.**
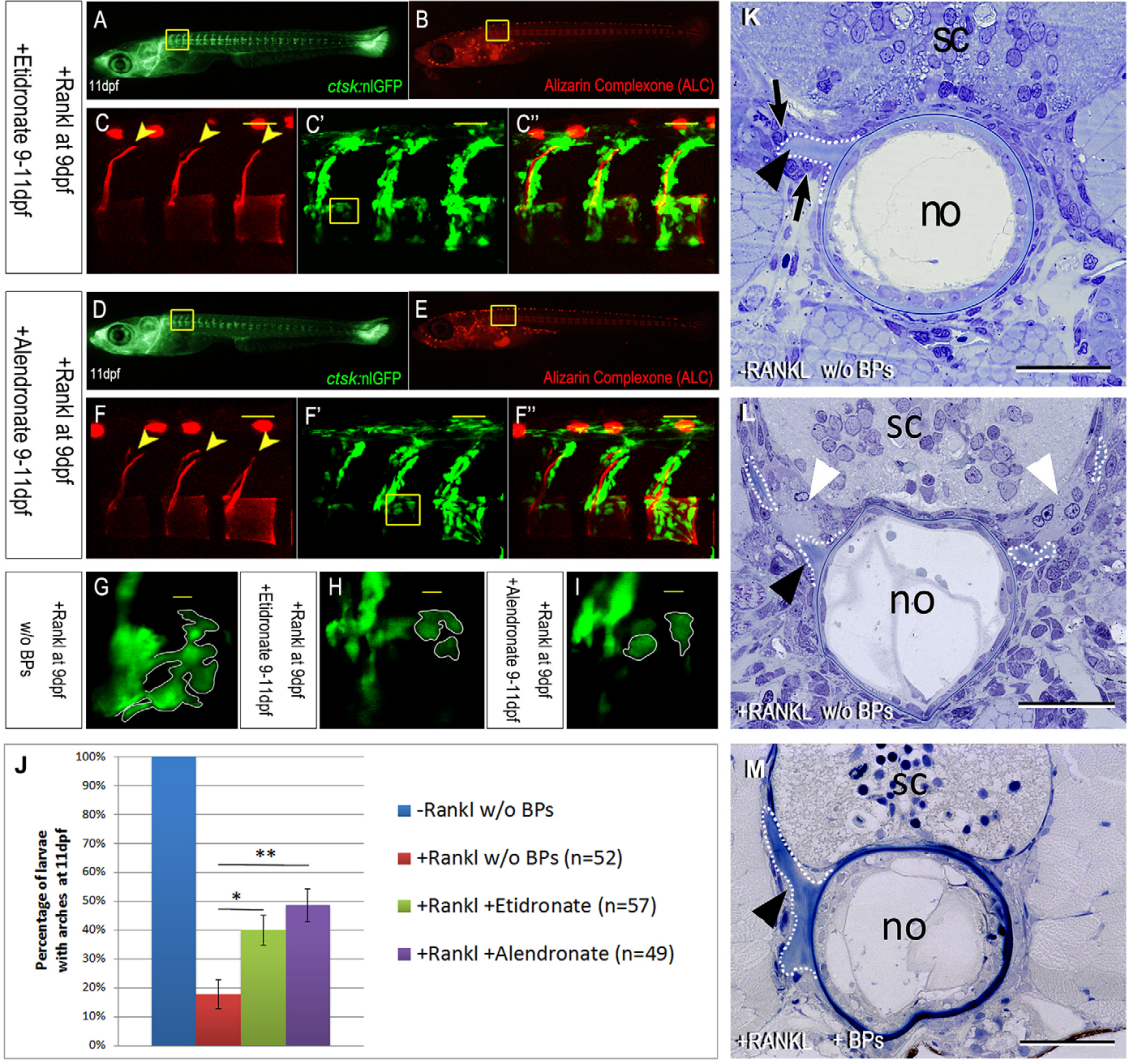
**Treatment with etidronate or alendronate blocks osteoclast function and bone resorption.** (A-C″) Expression of *ctsk*:nlGFP in ectopic osteoclasts after addition of etidronate on the same day as Rankl induction. (C-C″) Confocal imaging shows vertebral bodies with almost intact neural arches (arrowheads in C), in the presence of abundant *ctsk*:nlGFP-positive osteoclasts (C′). (D-F″) Expression of *ctsk*:nlGFP in osteoclasts after addition of alendronate on the same day as Rankl induction. (F-F″) Confocal imaging shows intact vertebral bodies with neural arches (arrowheads in F), in the presence of ectopic osteoclasts (F′). (G) Confocal image of *ctsk*:nlGFP-expressing osteoclasts after Rankl induction without BP treatment (taken from the same larvae as in Fig. 1F′, boxed area). (H, I) Confocal image of Rankl-induced osteoclasts after etidronate or alendronate treatment, respectively (taken from the same larvae shown in C′ and F′, boxed areas). (J) Statistical analysis of larvae with intact neural arches at 11 dpf, without Rankl induction (blue), after Rankl induction (red), after Rankl induction with etidronate (green; *0.01<*P*<0.05) or alendronate (purple; **P<0.01) treatment. (K-M) Transverse plastic sections (2 µm) through the notochord (no), spinal cord (sc) and the basis of the anteriormost neural arches at the same cross-sectional level, in −Rankl control larvae (K) and in larvae after Rankl induction without (L) or with (M) BP treatment. The base of the neural arches is labeled with a black arrowhead, osteoblasts are marked by black arrows and osteoclasts by white arrowheads. The basis of the bony arches is outlined by a dotted line. The sections in K,M are slightly oblique so that the neural arches of only one side can be seen. Scale bars: 50 μm in C,F; 30 µm in K-M.

Next, transgenic larvae were treated with etidronate and alendronate (Fig. 2). Treatment started on the same day as the heat shock (9 dpf) and continued for 2 days. BP treatment did not affect overall osteoclast formation (Fig. 2A,D). However, in 56.3% of BP-treated larvae the osteoclast distribution appeared reduced, especially in axial skeleton regions (see Fig. S3). This raises the possibility that BPs cause a delay in either formation or maturation of induced osteoclasts. Larvae with an equal extent of ectopic osteoclast formation were then analyzed after BP treatment for mineralization of the neural arches and vertebral centra (Fig. 2B,C,E,F) and compared with Rankl-induced larvae without BP treatment (Fig. 1E,F). The number of larvae with mineralization defects significantly decreased after treatment with etidronate (60.0%, *n*=57; Fig. 2C,J) and alendronate (51.5%, *n*=49; Fig. 2F,J). These rescued larvae showed almost completely intact neural arches and centra in the presence of ectopic osteoclasts (Fig. 2C-C″,F-F″). Notably, the remaining larvae showed mineralization defects that were qualitatively less severe than those observed in non-BP-treated +Rankl larvae (Fig. S4). Also, the general morphology of osteoclasts appeared to change after BP treatment. When compared with cells without BP treatment (Fig. 2G), cells after BP treatment often appeared less extended and more compacted (Fig. 2H,I). In histological sections of −Rankl control larvae, the basis of neural arches was surrounded by active osteoblasts, with no indication of resorption (Fig. 2K). In the +Rankl non-BP-treated group, larvae with severe vertebral defects showed areas where neural arches were resorbed and fenestrated by what appeared to be multinucleated osteoclasts (Fig. 2L, white arrowheads), leading to the absence of neural arches in ALC-stained specimens (Fig. 1F). In BP-treated animals, by contrast, intact arches were seen histologically (Fig. 2M), confirming the observations shown with ALC staining in Fig. 2C,F. Etidronate also prevented mineralization defects in arches when Rankl was induced at a much later time point (15 dpf; Fig. S5). These findings strongly suggest that treatment with etidronate and alendronate efficiently interferes with osteoclast function after induction by transgenic Rankl expression.

### Bisphosphonates induce morphological changes in medaka osteoclasts

**Fig. 3.**
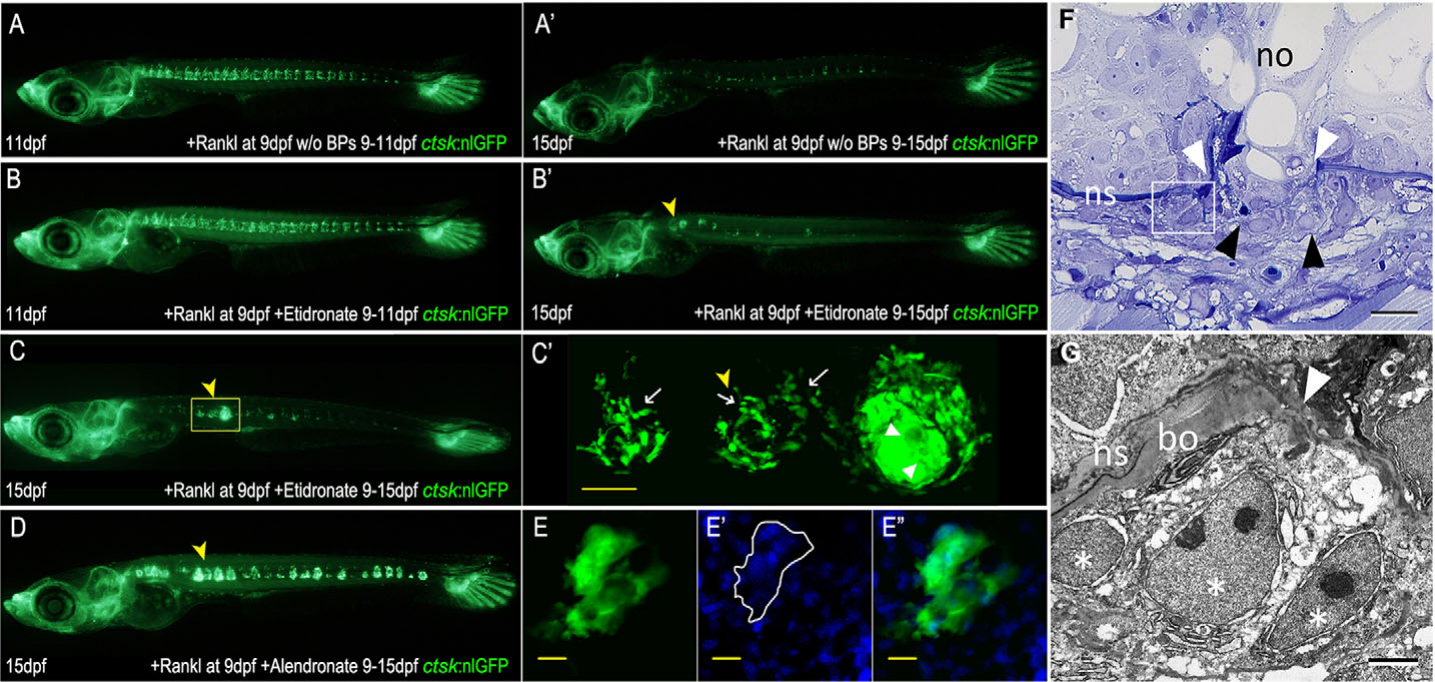
**BPs induce morphological changes in osteoclasts.** (A,A′) *ctsk*:nlGFP expression in one *rankl*:HSE:CFP/*ctsk*:nlGFP larva at 11 and 15 dpf, 2 and 6 days after Rankl induction, respectively. Note the reduction in the number of nlGFP-positive cells at 15 dpf. (B,B′) *ctsk*:nlGFP expression in one larva at 11 and 15 dpf, after Rankl induction and etidronate treatment. Note aggregation of nlGFP-positive cells (arrowhead). (C) *ctsk*:nlGFP expression in larvae at 15 dpf, after etidronate treatment. (C′) Confocal stack of area boxed in C showing accumulation of small osteoclasts (left and middle; arrows) and giant *ctsk*:nlGFP-expressing cell (right). (D) Larva with extensive aggregates of *ctsk*:nlGFP-expressing cells after alendronate treatment. (E-E″) *ctsk*:nlGFP-expressing cell aggregates stained with DAPI. (F) Ventral lesion (white arrowheads) in the notochord (no) after Rankl induction and BP treatment at 15 dpf (2 µm parasagittal plastic section). The notochordal sheath (ns) is disrupted, and notochordal cells are bulging out. They are delimited by a thin layer of matrix (black arrowheads). Outside, multiple nuclei are visible. (G) A higher magnification of this region in transmission electron microscopy shows that at least some nuclei (white asterisks) appear to belong to a single cell as they appear not to be separated by cell membranes. They adjoin the bone layer (bo) deposited outside the notochordal sheath (ns). Scale bars: 50 μm in C′; 20 μm in E; 15 µm in F; 2 µm in G.

When Rankl expression is triggered by heat shock at 9 dpf, the induced *ctsk*:nlGFP-expressing osteoclasts usually persist for several days (Fig. 3A), but most of the cells have disappeared at 15 dpf (To et al., 2012; Fig. 3A′). By contrast, we observed that a considerable fraction of etidronate-treated and alendronate-treated embryos continued to show *ctsk*:nlGFP-positive osteoclasts (*n*=7/95, 7.4% for etidronate and *n*=15/118, 12.7% for alendronate; Fig. 3C,D). These cells aggregated into large clusters and appeared to fuse into giant multinucleated cells (Fig. 3B′-E″; DAPI stain in Fig. 3E′,E″). Light and transmission electron microscopic observations confirmed the occurrence of cell aggregates and the possible presence of multinucleated cells within these aggregates (Fig. 3F,G). Similar cell behavior, i.e. aggregation and possible fusion, was not observed in *rankl*:HSE:CFP/*ctsk*:EGFP double transgenic medaka larvae without BP treatment (Fig. 3A′). This suggests that BPs can extend the lifetime of osteoclasts in medaka and possibly promote cell fusion. Interestingly, ‘giant-osteoclasts’ with extensively increased cell size and excess nuclei have been described in human osteoporosis patients after long-term BP treatment (Jobke et al., 2014). Thus, BPs can trigger effects in medaka that are similar to those observed in human patients.

### Bisphosphonate treatment stimulates bone recovery in medaka

**Fig. 4.**
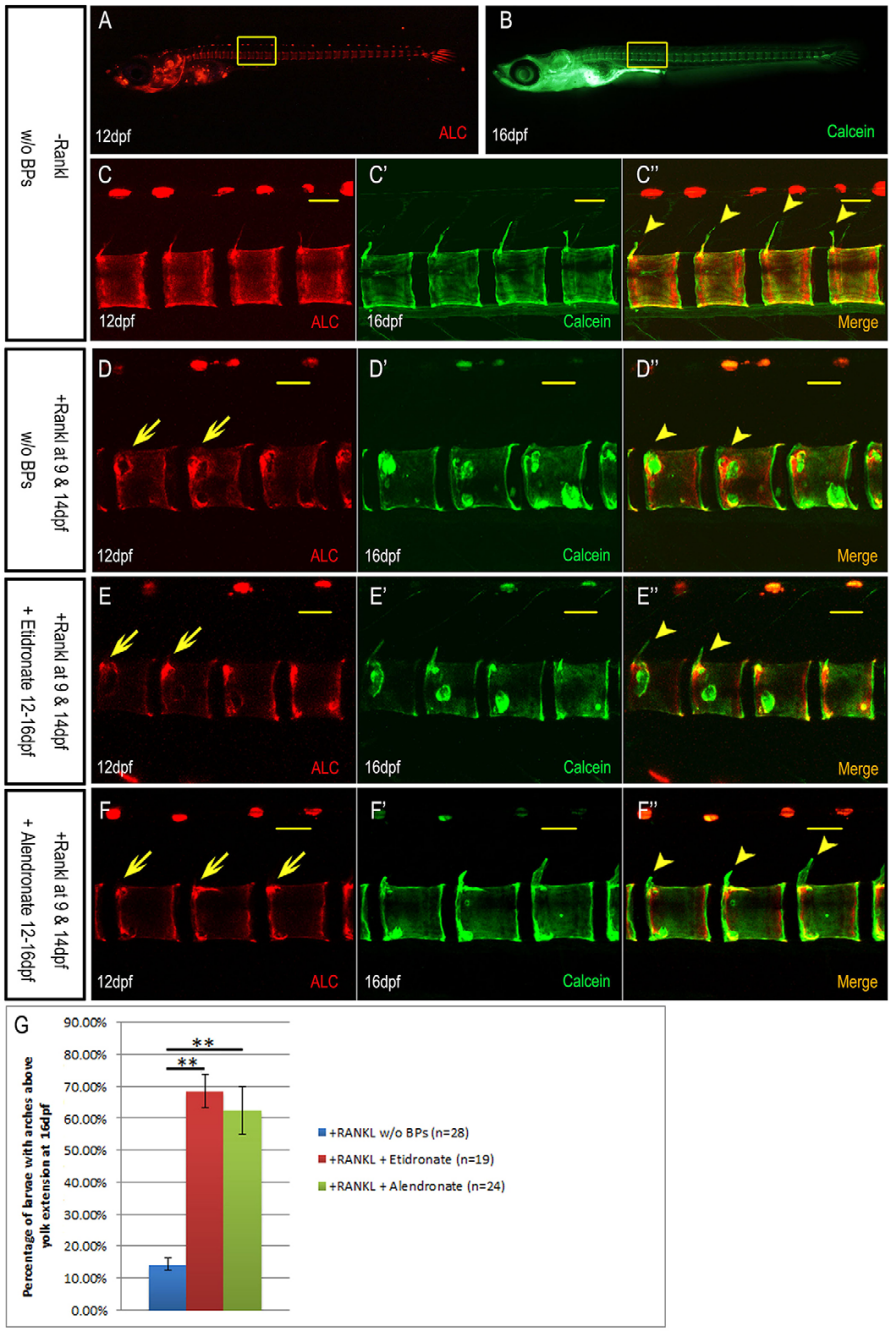
**Etidronate and alendronate accelerate bone recovery after blocking bone resorption in *rankl*:HSE:CFP larvae.** (A,B) Normal development of centra and arches from 12 dpf (ALC stained) to 16 dpf (calcein stained). (C-C″) Confocal stack of area boxed in (A,B), showing *de novo* mineralization at the tip of neural arches (arrowheads; calcein stained; C″) and around the notochordal sheath. (D-F) ALC-stained bone matrix at 12 dpf, 3 days after Rankl induction. Note lesions in the neural arches and centra (arrows). A second heat shock was carried out at 14 dpf. (D′-F′) At 16 dpf, the same larvae were stained with calcein to visualize newly formed mineralized bone matrix without (D-D″) and with etidronate (E-E″) or alendronate (F-F″) treatment. At this stage, lesions in centra are repaired (D-F), but neural arches are remineralized only after BP treatment (arrowheads; E″,F″) and not without BP treatment (D″). (G) Statistical analysis showing the percentage of larvae with neural arches at 16 dpf, after Rankl induction (blue), and after Rankl induction and etidronate (red) or alendronate (green) treatment. **P<0.01. Scale bars: 50 μm.

We next wanted to test whether BP treatment also affects bone remineralization. For this, we started BP treatment 3 days after osteoclasts had been induced by transgenic Rankl expression. In a similar manner to the previous experiment, *rankl*:HSE:CFP transgenic larvae were subjected to heat shock at 9 dpf, except that the heat shock was extended to 2 h. In these conditions, ectopic osteoclasts are efficiently induced and more than 90% of the larvae exhibit a complete loss of mineralized neural arches at 12 dpf when compared with non-heat-shocked controls (data not shown). We then started BP treatment in larvae with strong mineralization defects at 12 dpf, as analyzed by ALC staining. Larvae were subjected to a second heat shock at 14 dpf to induce severe lesions in the centra. The second heat shock was performed to enhance the effect of Rankl induction, and thereby, make the effect of BP treatment more obvious. BP treatment was done from 12 to 16 dpf (i.e. 3 days after osteoclast induction) in order to detect any effects on bone recovery (Fig. 4). *De novo* mineralization was analyzed by successively staining larvae with calcein at 16 dpf. This allows previously existing mineralized (stained with ALC, red) matrix to be distinguished from *de novo* mineralized matrix (stained with calcein, green). Accordingly, in non-heat-shocked control embryos without BP treatment, newly mineralized matrix could be detected at the tips of the extending neural arches and around the notochordal sheath (Fig. 4A-C″). For +Rankl non-BP-treated control larvae, repair of lesions in the centra region can be observed, but only 14.3% of heat-shocked embryos developed neural arches (Fig. 4D-D″). By contrast, with BP treatment, 68.4% larvae after etidronate (Fig. 4E-E″,G) and 62.5% after alendronate (Fig. 4F-F″,G) treatment showed partly recovered neural arches at 16 dpf. Compared with arches in −Rankl non-BP-treated control larvae (Fig. 4C″), the arches in BP-treated larvae were composed exclusively from *de novo* mineralized matrix as evident by uniform calcein staining in absence of any ALC label (Fig. 4E″,F″). These findings suggest that in medaka, BPs stimulate bone recovery by blocking osteoclast function.

In severe cases, +Rankl non-BP-treated control larvae showed newly mineralized matrix only at the edges of the lesions induced in centra (Fig. S6A-A″). By contrast, larvae after etidronate and alendronate treatment showed an almost complete *de novo* remineralization of cavities in the centra (Fig. S6B″-C″). This suggests that after blocking osteoclast activity in medaka by BPs, coexisting osteoblasts efficiently remineralize damaged bone matrix, resulting in bone recovery.

### Bisphosphonate treatment affects osteoblast distribution

**Fig. 5.**
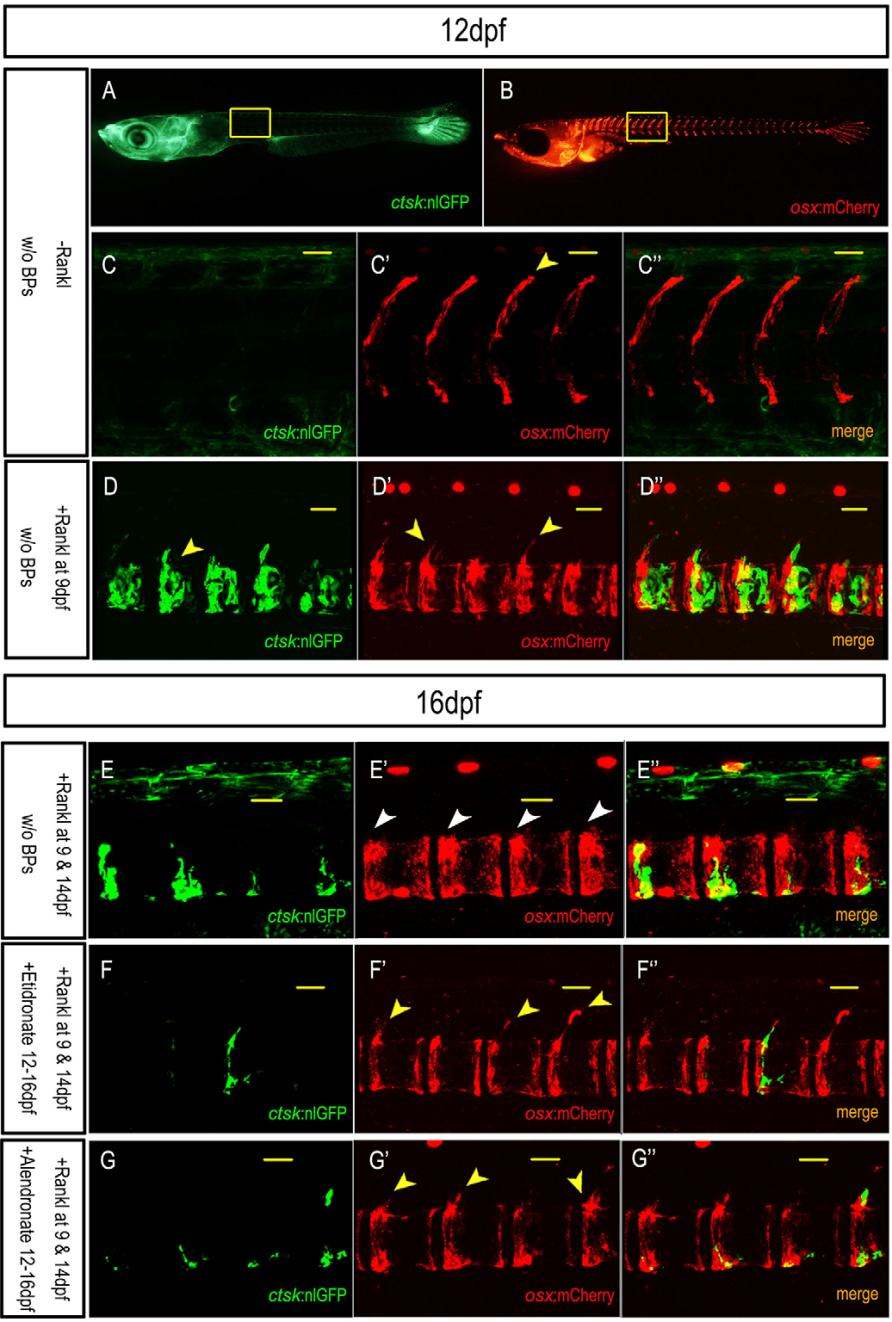
**Effect of BP treatment on osteoblast distribution.** (A-C) Expression of *osx*:mCherry in osteoblasts without Rankl induction. (C-C″) Confocal stack of area boxed in (A,B), showing absence of *ctsk*:nlGFP-expressing osteoclasts in vertebral bodies at 12 dpf (C). *osx*:mCherry-positive osteoblasts are positioned along neural arches and the edges of centra (arrowhead; C′). (D-D″) Position of *osx*:mCherry-positive osteoblasts after ectopic osteoclast induction. Confocal stack showing *ctsk*:nlGFP cells in vertebral bodies of Rankl-induced larvae (arrowhead; D). Note reduction of *osx*:mCherry-positive osteoblasts around arches and increased presence in centra (arrowheads; D′). A second heat shock was performed at 14 dpf. (E-G″) Osteoblast-osteoclast behavior was observed at 16 dpf, with Rankl induction and no BP treatment (E-E″) and with both Rankl induction and etidronate (F-F″) or alendronate (G-G″) treatment. The number of osteoclasts is reduced at 16 dpf (E,F,G) when compared with 12 dpf (D). Note the reduced numbers of *osx*:mCherry-positive cells around neural arches but increased numbers in the centra (arrowheads; E′) without BP treatment. By contrast, more *osx*:mCherry-positive cells are positioned along neural arches (arrowheads; F′,G′) after etidronate (F-F″) or alendronate (G-G″) treatment from 12 to 16 dpf. This coincides with reduced numbers of *osx*:mCherry-positive cells in centra. Scale bars: 50 μm.

Osteoblast-osteoclast coupling implies a tightly coordinated interaction between both cell types in order to maintain appropriate cell numbers and bone homeostasis. In human osteoporosis patients, long-term BP treatment reduces bone formation by osteoblasts, but the underlying cellular mechanisms remain unclear (reviewed by Charles and Aliprantis, 2014). We therefore tested whether osteoblast-osteoclast coupling is affected by BP treatment in medaka. Specifically, we assessed whether the behavior of osteoblasts expressing *osterix* (*osx*) is altered when larvae are treated with BPs. For this, we subjected *rankl*:HSE:CFP/*ctsk*:nlGFP/*osx*:mCherry triple transgenic medaka larvae to two successive rounds of heat shock at 9 and 14 dpf and assessed formation and distribution of *osx*-expressing osteoblasts with and without BP treatment. As controls, neither heat shock alone (without Rankl) nor BP treatment alone affected distribution of *osx*:mCherry-positive osteoblasts (Fig. S7). Significant differences in the distribution of *osx*-positive osteoblasts were observed between larvae without (Fig. 5A-C) and with ectopic osteoclast formation (Fig. 5D-D″) at 12 dpf (see also Renn et al., 2013). The number of *osx*-positive cells was strongly reduced along the neural arches, and instead, *osx*-positive cells accumulated in the centra (Fig. 5D′) along with the presence of active osteoclasts (Fig. 5D″). At 16 dpf, osteoblasts were completely absent from the neural arches in Rankl-induced larvae without BP treatment and were only found in the centra (Fig. 5E′-E″). By contrast, in larvae treated with BPs from 12 to 16 dpf, osteoblasts were present along the neural arches and were less prominent in the centra (Fig. 5F′,G′). Intriguingly, osteoclasts were abundant in these embryos with Rankl expression (Fig. 5F,G). Together, these results suggest that Rankl-induced, active osteoclasts drive osteoblasts to lesion sites inside the centra. In the presence of BPs, however, many osteoblasts remain at the arches and contribute to their mineralization.

## DISCUSSION

Zebrafish and medaka have become popular models in bone research. They complement each other and offer a unique combination of large-scale genetics with live imaging to identify and characterize novel factors involved in bone formation and remodeling (Mackay et al., 2013). Although the zebrafish has cellular bones containing osteocytes and offers a large number of well-characterized bone mutants (Apschner et al., 2014; Mackay et al., 2013), the medaka is characterized by acellular bone without osteocytes and provides many useful transgenic bone reporter lines (Takeyama et al., 2014; To et al., 2012). The almost transparent fish embryos and larvae allow visualization of cellular processes by live imaging that are not accessible in mammalian model systems. Large numbers of fish embryos and larvae can easily be obtained on a daily basis and maintained in minimal volumes of simple media. This makes them ideal models for high-content and low-cost screening of clinically relevant compounds (for review see Ablain and Zon, 2013). Despite these obvious advantages, fish models have rarely been used so far to characterize the effect of bone-anabolic or antiresorptive drugs on osteoblasts and osteoclasts *in vivo*.

We previously reported two transgenic medaka osteoporosis models. In the first model, the number of osteoblasts was reduced by conditional cell ablation, leading to reduced bone formation (Willems et al., 2012). In the second model, osteoclast formation was induced by ectopic Rankl expression, leading to increased bone resorption and reduced mineralization of bones and the notochordal sheath (To et al., 2012). In the present study, we used this latter model to test the effects of BPs on osteoclast formation and activity. Our data show that treatment with etidronate or alendronate prevents bone loss in transgenic medaka that is caused by ectopic osteoclasts and stimulates bone recovery by blocking the bone-resorptive function of osteoclasts. We found that Rankl induction resulted in an upregulation of tumor necrosis factor-α expression, thus suggesting the trigger of an inflammatory response (Fig. S8). Interestingly, this response was attenuated in the presence of BPs, raising the possibility that BPs modulate inflammatory reactions during increased bone resorption.

In preclinical models and human patients, alendronate was described to be significantly more potent than etidronate (Iwamoto et al., 2005; Masarachia et al., 1996). In the present medaka study, however, both BPs appeared to have similar efficacy in blocking osteoclast activity and stimulating bone recovery; in fact, with a higher effective dose of alendronate compared with etidronate. The reason for this difference remains unknown but possibly reflects biochemical differences of the affected pathways in larval compared with adult osteoclasts. Alternatively, the possibilities cannot be excluded that BPs bind different targets in teleosts compared with humans or that common targets are poorly conserved.

Bone resorption requires a sealed extracellular microenvironment and tight contact between osteoclasts and the mineralized matrix in order to generate resorption lacunae and allow proper osteoclast function. Using live confocal imaging, we observed osteoclasts in the vicinity of mineralized neural arches and vertebral bodies after BP treatment. However, these osteoclasts failed to resorb mineralized matrix. We speculate that these osteoclasts were probably not capable of generating sealed lacunae required for resorption. Future histological analyses will show the morphological defects induced in BP-treated medaka osteoclasts.

After long-term treatment with etidronate or alendronate, changes in osteoclast morphology were described in osteoporosis patients of different ages and sexes. Multinucleated osteoclasts became non-functional and underwent cell fusion, leading to ‘giant’ osteoclasts with a two- to threefold increase in the number of nuclei (Jobke et al., 2014; Weinstein et al., 2009). Similar observations were made in the medaka model, where osteoclasts after BP treatment showed reduced resorptive activity but extended lifespan and appeared to aggregate and even fuse into multinucleated cells. Together, our observations that BPs block osteoclast activity in medaka and stimulate the formation of multinucleated osteoclasts with increased size faithfully recapitulate aspects found in human osteoporosis patients. This therefore makes the transgenic medaka described here an efficient and valuable *in vivo* model for future drug screening to identify novel antiresorptive and bone-anabolic compounds.

We found that BP treatment did not affect general induction of osteoclasts after transgenic Rankl induction. However, we observed a generally less extensive distribution of osteoclasts in 56.3% of the BP-treated larvae when heat shock and BPs were applied at the same stage at 9 dpf. When BP treatment was started 1-4 days before Rankl induction, the effect on osteoclast formation was even more pronounced (Fig. S9). It remains to be shown whether this is the result of a reduction in osteoclast numbers or a possible delay in morphological maturation. Our observation raises the possibility that osteoclast maturation depends on a proper interaction of osteoclast precursors with mineralized matrix, which could be impaired by matrix-bound BPs. In human BP research, focus has been placed mainly on the effect of BPs on the inhibition of osteoclast resorption and survival. Much less attention has been paid to understanding the effects of BP on early osteoclast differentiation and maturation. Our *in vivo* observations suggest that these early differentiation stages deserve further investigation, especially in the context of therapeutic use.

The availability of transgenic medaka lines expressing multiple fluorescent reporters allows simultaneous visualization of different cell types in experimental conditions such as increased bone resorption or BP treatment. This is especially useful to characterize osteoblast-osteoclast coupling in an *in vivo* context. Interestingly, we found that the distribution of *osx*-positive osteoblasts was altered after BP treatment and that these osteoblasts contributed to an efficient bone repair. This suggests that osteoclast signaling to maintain osteoblast numbers and distribution is affected by the presence of BPs, at least on a short-term basis (see model in Fig. S10). Future studies need to show whether this also persists after long-term BP treatment and whether the osteogenic activity of osteoblasts is affected. We have previously shown that Rankl-induced osteoclastogenesis and subsequent resorption of the mineralized matrix triggers a remineralization process that involves recruitment of osteoblast progenitors to the lesion sites (Renn et al., 2013). It will be interesting to test in detail in future experiments whether this osteoblast progenitor recruitment is affected by BP treatment, which would have major implications for antiresorptive therapies in human osteoporosis patients. Our most striking observation was that BP treatment in medaka stimulated *de novo* mineralization of neural arches and vertebral bodies. This suggests the presence of active osteoblasts in the vertebral column that are able to repair bone lesions as soon as osteoclast activity is blocked by BPs. Whether this reflects the enormous regenerative potential of teleost fish or can also be found in defective mammalian bone remains to be elucidated in future experiments.

In mammals, BPs suppress bone formation and act as potent mineralization inhibitors (reviewed by Charles and Aliprantis, 2014). Surprisingly, in our studies, we observed accelerated bone recovery and rapid remineralization of bone matrix after BP treatment in medaka, which would suggest anabolic effects of BPs in this model. At present, we cannot explain why BPs in medaka allow efficient remineralization of bone lesions. Future studies using live imaging in transparent medaka larvae in different mineralization conditions need to uncover the mechanisms underlying the anabolic effects of BPs in this system.

Osteoporosis is a disorder primarily affecting people older than 50 years of age, and in particular, postmenopausal women (Charles and Aliprantis, 2014). It would therefore be interesting to test the effect of BPs on older medaka fish of both sexes, in which osteoclasts have been activated during adult stages. Different from the zebrafish, the medaka has heteromorphic sex chromosomes, thus allowing a sex-specific analysis during larval and adult stages (Nanda et al., 2002). Furthermore, bone defects have been reported in adult medaka in a sex-specific manner (Shanthanagouda et al., 2014). Therefore, the medaka model is excellently suited to study the biology behind age- and sex-specific bone remodeling and to test the efficiency of potential therapeutics. In conclusion, the transgenic medaka model described in the present study provides unique and novel insight into the cell interactions taking place during bone remodeling, BP treatment and bone repair.

## MATERIALS AND METHODS

### Maintenance of transgenic fish

Wild-type, *rankl*:HSE:CFP, *ctsk*:nlGFP and *osx*:mCherry single or compound transgenic medaka fish were kept at 26°C under a controlled light cycle (14 h light, 10 h dark) to induce spawning. Embryos were kept in 0.3× Danieau's solution [19.3 mM NaCl, 0.23 mM KCl, 0.13 mM MgSO_4_, 0.2 mM Ca(NO_3_)_2_ and 1.7 mM HEPES, pH 7.0] at 30°C, and medium was changed daily to ensure normal development of the embryos. Embryos were staged according to Iwamatsu (2004). Embryos were screened for fluorescence reporter expression from 5 dpf onwards. All experiments were performed in accordance with approved IACUC protocols of the National University of Singapore (R14-293).

### Bisphosphonate treatment of fish larvae

As described previously (To et al., 2012), *rankl*:HSE:CFP transgenic medaka larvae at 9 dpf were subjected to heat shock at 39°C for 1.5 or 2 h to induce Rankl expression efficiently. After a recovery period of 1 h at 30°C, larvae were screened for CFP expression indicating successful Rankl induction. Larvae showing expression of both CFP and *ctsk*:nlGFP were transferred to six-well plates (six larvae per well) for subsequent BP treatment. Etidronate (Sigma P5248; 5, 7.5, 10, 12.5 and 15 µg/ml) and alendronate (Sigma A4978; 25, 37.5, 50, 62.5 and 75 µg/ml) were dissolved in fish medium and added to the larvae at different time points after induction of heat shock, as specified in the Results section. Control larvae were kept in fish medium after heat shock (+Rankl non-BP-treated control) or received no heat shock (−Rankl non-BP-treated control; −Rankl BP-treated control). All media were changed daily.

### Live staining of mineralized matrix

For bone matrix staining, medaka larvae were incubated in 0.1% alizarin-3-methyliminodiacetic acid (alizarin complexone, ALC; Sigma A3882) or 0.01% calcein (Sigma C0875) in fish medium at 30°C for 1.5 or 2.5 h (for larvae at 9-17 dpf). After incubation, larvae were rinsed in fish medium for 30 min to 1 h before being mounted for imaging. To ensure that all larvae were at the same developmental stage for BP treatment, ALC staining was conducted to count mineralized caudal fin rays. Staining was analyzed using tetramethylrhodamine (TRITC) and GFP filter settings. Larvae with four mineralized caudal fin rays (equivalent to 9 dpf) were selected for further analysis.

### Imaging

For live fluorescence imaging, larvae were anesthetized with 0.01% ethyl 3-aminobenzoate methanesulfonate (tricaine; Sigma A5040) and pictures were taken using a Nikon SMZ1000 stereomicroscope equipped with NIS-Elements BR 3.0 software (Nikon, Japan). For live confocal imaging, larvae were anesthetized with 0.005% tricaine and embedded in 1.5% low-melting-point agarose in a glass-bottomed Petri dish. Confocal pictures were taken with a Zeiss LSM 510 Meta using 405, 488 and 543 nm laser lines for DAPI, GFP and mCherry analysis, respectively. Imaging data were processed using Zeiss LSM Image Browser Version 4.2.0.121, Imaris 7.1.1 (bitplane), Image-Pro Plus 6.0, ImageJ (1.4.3.67) and Adobe Photoshop CS6 (13.0.0.0) software.

### Reverse transcription (RT)-PCR analysis

Twenty larvae at 11 dpf were used for RNA extraction using the RNeasy Mini Kit (Qiagen 74104). Larvae without Rankl induction and BP treatment served as controls. All RNA samples were subjected to DNase I digestion. RNA was reverse transcribed using the RevertAid First Strand cDNA Synthesis Kit (Life Technologies K1621). *β-actin* was used for normalization. The fold up- or downregulation of relative gene expression levels was calculated from three biological replicates using ImageJ. The following primers were used (forward, reverse): *β-actin* (5′-TTCAACAGCCCTGCCATGTA-3′, 5′-GCAGCTCATAGCTCTTCTCCAGGGAG-3′); and *TNF-α* (5′-GGAAGATACTTGTGGTCCTGGTCT-3′, 5′-CCTCCCACTGATTTTGAGAAGC-3′).

### Statistical analysis

The number of larvae with the indicated phenotype was recorded. Results are presented as percentages with mean±s.d. as determined using Excel. Two-tailed Student's *t*-test was used for comparison of groups individually and determination of significance. The level of significance was set as follows: *0.01<*P*<0.05 and **P<0.01.

### Histological analysis

For plastic sections and transmission electron microscopy, larvae were either fixed in 10% formalin (Fig. 2L,M; Fig. 3F,G) or in a mixture of 1.5% paraformaldehyde and 1.5% glutaraldehyde in 0.1 M sodium cacodylate buffer (pH.7.4; Fig. 2K) for a minimum of 2 h at room temperature. Larvae were post-fixed with osmium tetroxide [1.25 ml OsO_4_, 2.5 ml sodium cacodylate buffer 0.2 M (pH 7.4), 0.4 g saccharose, 0.04 ml CaCl_2_ (0.5%) and distilled H_2_O to make up to 5 ml] and embedded in Epon epoxide medium. Semithin sections (2 µm) were stained with toluidine blue. Ultrathin sections were cut on a Reichert-OM U3 ultramicrotome, mounted on coated, single-hole copper grids and contrasted with uranyl acetate and lead citrate.

## References

[DMM019091C1] Ablain J. and Zon L. I. (2013). Of fish and men: using zebrafish to fight human diseases. *Trends Cell Biol.* 23, 584-586. 10.1016/j.tcb.2013.09.009 24275383 PMC6047759

[DMM019091C2] Apschner A., Schulte-Merker S. and Witten P. E. (2011). Not all bones are created equal - using zebrafish and other teleost species in osteogenesis research. *Methods Cell Biol.* 105, 239-255. 10.1016/B978-0-12-381320-6.00010-2 21951533

[DMM019091C3] Apschner A., Huitema L. F. A., Ponsioen B., Peterson-Maduro J. and Schulte-Merker S. (2014). Zebrafish enpp1 mutants exhibit pathological mineralization, mimicking features of generalized arterial calcification of infancy (GACI) and pseudoxanthoma elasticum (PXE). *Dis. Model. Mech.* 7, 811-822. 10.1242/dmm.015693 24906371 PMC4073271

[DMM019091C4] Barrett R., Chappell C., Quick M. and Fleming A. (2006). A rapid, high content, in vivo model of glucocorticoid-induced osteoporosis. *Biotechnol. J.* 1, 651-655. 10.1002/biot.200600043 16892313

[DMM019091C5] Benford H. L., McGowan N. W. A., Helfrich M. H., Nuttall M. E. and Rogers M. J. (2001). Visualization of bisphosphonate-induced caspase-3 activity in apoptotic osteoclasts in vitro. *Bone* 28, 465-473. 10.1016/S8756-3282(01)00412-4 11344045

[DMM019091C6] Charles J. F. and Aliprantis A. O. (2014). Osteoclasts: more than ‘bone eaters’. *Trends Mol. Med.* 20, 449-459. 10.1016/j.molmed.2014.06.001 25008556 PMC4119859

[DMM019091C7] Dominguez L. J., Di Bella G., Belvedere M. and Barbagallo M. (2011). Physiology of the aging bone and mechanisms of action of bisphosphonates. *Biogerontology* 12, 397-408. 10.1007/s10522-011-9344-5 21695491

[DMM019091C8] Ebetino F. H., Hogan A.-M., Sun S., Tsoumpra M. K., Duan X., Triffitt J. T., Kwaasi A. A., Dunford J. E., Barnett B. L., Oppermann U. et al. (2011). The relationship between the chemistry and biological activity of the bisphosphonates. *Bone* 49, 20-33. 10.1016/j.bone.2011.03.774 21497677

[DMM019091C9] Fleming A., Sato M. and Goldsmith P. (2005). High-throughput in vivo screening for bone anabolic compounds with zebrafish. *J. Biomol. Screen.* 10, 823-831. 10.1177/1087057105279952 16234346

[DMM019091C10] Harada S.-I. and Rodan G. A. (2003). Control of osteoblast function and regulation of bone mass. *Nature* 423, 349-355. 10.1038/nature01660 12748654

[DMM019091C29] Iwamatsu T. (2004). Stages of normal development in the medaka *Oryzias latipes*. *Mech. Dev.* 121, 605-618. 10.1016/j.mod.2004.03.012 15210170

[DMM019091C11] Iwamoto J., Takeda T., Sato Y. and Uzawa M. (2005). Comparison of effect of treatment with etidronate and alendronate on lumbar bone mineral density in elderly women with osteoporosis. *Yonsei Med. J.* 46, 750-758. 10.3349/ymj.2005.46.6.750 16385649 PMC2810587

[DMM019091C12] Jobke B., Milovanovic P., Amling M. and Busse B. (2014). Bisphosphonate-osteoclasts: changes in osteoclast morphology and function induced by antiresorptive nitrogen-containing bisphosphonate treatment in osteoporosis patients. *Bone* 59, 37-43. 10.1016/j.bone.2013.10.024 24211427

[DMM019091C13] Khosla S. and Riggs B. L. (2005). Pathophysiology of age-related bone loss and osteoporosis. *Endocrinol. Metab. Clin. North Am.* 34, 1015-1030, xi 10.1016/j.ecl.2005.07.009 16310636

[DMM019091C14] Luckman S. P., Hughes D. E., Coxon F. P., Graham R., Russell G. and Rogers M. J. (1998). Nitrogen-containing bisphosphonates inhibit the mevalonate pathway and prevent post-translational prenylation of GTP-binding proteins, including Ras. *J. Bone Miner. Res.* 13, 581-589. 10.1359/jbmr.1998.13.4.581 9556058

[DMM019091C15] Mackay E. W., Apschner A. and Schulte-Merker S. (2013). A bone to pick with zebrafish. *Bonekey Rep.* 2, 445 10.1038/bonekey.2013.179 24422140 PMC3844975

[DMM019091C16] Manolagas S. C. (2010). From estrogen-centric to aging and oxidative stress: a revised perspective of the pathogenesis of osteoporosis. *Endocr. Rev.* 31, 266-300. 10.1210/er.2009-0024 20051526 PMC3365845

[DMM019091C17] Masarachia P., Weinreb M., Balena R. and Rodan G. A. (1996). Comparison of the distribution of 3H-alendronate and 3H-etidronate in rat and mouse bones. *Bone* 19, 281-290. 10.1016/8756-3282(96)00182-2 8873969

[DMM019091C18] Nanda I., Kondo M., Hornung U., Asakawa S., Winkler C., Shimizu A., Shan Z., Haaf T., Shimizu N., Shima A. et al. (2002). A duplicated copy of DMRT1 in the sex-determining region of the Y chromosome of the medaka, Oryzias latipes. *Proc. Natl. Acad. Sci. USA* 99, 11778-11783. 10.1073/pnas.182314699 12193652 PMC129345

[DMM019091C19] Nanes M. S. and Kallen C. B. (2009). Clinical assessment of fracture risk and novel therapeutic strategies to combat osteoporosis. *Fertil. Steril.* 92, 403-412. 10.1016/j.fertnstert.2009.05.049 19559412

[DMM019091C20] Renn J. and Winkler C. (2009). Osterix-mCherry transgenic medaka for in vivo imaging of bone formation. *Dev. Dyn.* 238, 241-248. 10.1002/dvdy.21836 19097055

[DMM019091C21] Renn J., Buttner A., To T. T., Chan S. J. H. and Winkler C. (2013). A col10a1:nlGFP transgenic line displays putative osteoblast precursors at the medaka notochordal sheath prior to mineralization. *Dev. Biol.* 381, 134-143. 10.1016/j.ydbio.2013.05.030 23769979

[DMM019091C22] Rogers M. J., Crockett J. C., Coxon F. P. and Monkkonen J. (2011). Biochemical and molecular mechanisms of action of bisphosphonates. *Bone* 49, 34-41. 10.1016/j.bone.2010.11.008 21111853

[DMM019091C23] Shanthanagouda A. H., Guo B.-S., Ye R. R., Chao L., Chiang M. W. L., Singaram G., Cheung N. K. M., Zhang G. and Au D. W. T. (2014). Japanese medaka: a non-mammalian vertebrate model for studying sex and age-related bone metabolism in vivo. *PLoS ONE* 9, e88165 10.1371/journal.pone.0088165 24523879 PMC3921145

[DMM019091C24] Takeyama K., Chatani M., Takano Y. and Kudo A. (2014). *In-vivo* imaging of the fracture healing in medaka revealed two types of osteoclasts before and after the callus formation by osteoblasts. *Dev. Biol.* 394, 292-304. 10.1016/j.ydbio.2014.08.007 25131195

[DMM019091C25] To T. T., Witten P. E., Renn J., Bhattacharya D., Huysseune A. and Winkler C. (2012). Rankl-induced osteoclastogenesis leads to loss of mineralization in a medaka osteoporosis model. *Development* 139, 141-150. 10.1242/dev.071035 22096076

[DMM019091C26] Weinstein R. S., Roberson P. K. and Manolagas S. C. (2009). Giant osteoclast formation and long-term oral bisphosphonate therapy. *N. Engl. J. Med.* 360, 53-62. 10.1056/NEJMoa0802633 19118304 PMC2866022

[DMM019091C27] Willems B., Buttner A., Huysseune A., Renn J., Witten P. E. and Winkler C. (2012). Conditional ablation of osteoblasts in medaka. *Dev. Biol.* 364, 128-137. 10.1016/j.ydbio.2012.01.023 22326228

[DMM019091C28] Witten P. E. and Huysseune A. (2009). A comparative view on mechanisms and functions of skeletal remodelling in teleost fish, with special emphasis on osteoclasts and their function. *Biol. Rev.* 84, 315-346. 10.1111/j.1469-185X.2009.00077.x 19382934

